# Conducting gender-based analysis of existing databases when self-reported gender data are unavailable: the GENDER Index in a working population

**DOI:** 10.17269/s41997-019-00277-2

**Published:** 2020-01-13

**Authors:** Anaïs Lacasse, M. Gabrielle Pagé, Manon Choinière, Marc Dorais, Bilkis Vissandjée, Hermine Lore Nguena Nguefack, Joel Katz, Oumar Mallé Samb, Alain Vanasse, on behalf of the TORSADE Cohort Working Group, Alain Vanasse, Gillian Bartlett, Lucie Blais, David Buckeridge, Manon Choinière, Catherine Hudon, Anaïs Lacasse, Benoit Lamarche, Alexandre Lebel, Amélie Quesnel-Vallée, Pasquale Roberge, Valérie Émond, Marie-Pascale Pomey, Mike Benigeri, Anne-Marie Cloutier, Marc Dorais, Josiane Courteau, Mireille Courteau, Stéphanie Plante, Pierre Cambon, Annie Giguère, Isabelle Leroux, Danielle St-Laurent, Denis Roy, Jaime Borja, André Néron, Geneviève Landry, Jean-François Ethier, Roxanne Dault, Marc-Antoine Côté-Marcil, Pier Tremblay, Sonia Quirion

**Affiliations:** 1 Département des sciences de la santé, Université du Québec en Abitibi-Témiscamingue (UQAT), 445, boul. de l’Université, Rouyn-Noranda, Québec J9X 5E4 Canada; 2 Centre de recherche du Centre hospitalier de l’Université de Montréal (CRCHUM), Montréal, Québec Canada; 3 Département d’anesthésiologie et de médecine de la douleur, Faculté de médecine, Université de Montréal, Montréal, Québec Canada; 4 StatSciences Inc., Notre-Dame-de-l’Île-Perrot, Québec Canada; 5 Faculté des sciences infirmières, Université de Montréal, Montréal, Québec Canada; 6 Institut de recherche en santé publique, Université de Montréal, Montréal, Québec Canada; 7 Department of Psychology, Faculty of Health, York University, Toronto, Ontario Canada; 8 Département de médecine de famille et de médecine d’urgence, Faculté de médecine et des sciences de la santé, Université de Sherbrooke, Sherbrooke, Québec Canada; 9 Centre de recherche du Centre hospitalier universitaire de Sherbrooke (CRCHUS), Sherbrooke, Québec Canada

**Keywords:** Sex, Gender, Composite index, Measurement, Administrative databases, Existing data, Secondary analysis, Canadian Community Health Survey, CCHS, Workers, Sexe, Genre, Indice composite, Mesure, Données administratives, Données existantes, Analyse secondaire, Enquête sur la santé dans les collectivités canadiennes, ESCC, Travailleurs

## Abstract

**Objectives:**

Growing attention has been given to considering sex and gender in health research. However, this remains a challenge in the context of retrospective studies where self-reported gender measures are often unavailable. This study aimed to create and validate a composite gender index using data from the Canadian Community Health Survey (CCHS).

**Methods:**

According to scientific literature and expert opinion, the GENDER Index was built using several variables available in the CCHS and deemed to be gender-related (e.g., occupation, receiving child support, number of working hours). Among workers aged 18–50 years who had no missing data for our variables of interest (*n* = 29,470 participants), propensity scores were derived from a logistic regression model that included gender-related variables as covariates and where biological sex served as the dependent variable. Construct validity of propensity scores (GENDER Index scores) were then examined.

**Results:**

When looking at the distribution of the GENDER Index scores in males and females, they appeared related but partly independent. Differences in the proportion of females appeared between groups categorized according to the GENDER Index scores tertiles (*p* < 0.0001). Construct validity was also examined through associations between the GENDER Index scores and gender-related variables identified a priori such as choosing/avoiding certain foods because of weight concerns (*p* < 0.0001), caring for children as the most important thing contributing to stress (*p* = 0.0309), and ability to handle unexpected/difficult problems (*p* = 0.0375).

**Conclusion:**

The GENDER Index could be useful to enhance the capacity of researchers using CCHS data to conduct gender-based analysis among populations of workers.

## Résumé

**Objectifs:**

Dans le domaine de la recherche en santé, une attention grandissante est portée à l’importance de tenir compte à la fois du sexe et du genre. Toutefois, ceci représente un défi quand on travaille avec des données existantes qui ne contiennent pas toujours de mesure autoraportée de genre. L’objectif de cette étude était donc de développer et valider un indice composite de genre grâce aux données de l’Enquête sur la santé dans les collectivités canadiennes (ESCC).

**Méthodologie:**

Basé sur la littérature et l’opinion d’experts, le GENDER Index a été développé grâce à plusieurs variables contenues dans l’ESCC et potentiellement liées au genre (ex. occupation, recevoir une pension alimentaire, nombre d’heures travaillées). Chez les travailleurs de 18 à 50 ans n’ayant pas de données manquantes sur le plan des variables sélectionnées (*n* = 29 470 participants), des scores de propension ont été dérivés d’un modèle de régression logistique dans lequel les variables potentiellement liées au genre ont été incorporées comme covariables et le sexe biologique a été considéré comme variable dépendante. La validité conceptuelle des scores de propension obtenus (scores du GENDER Index) a ensuite été explorée.

**Résultats:**

Sur le plan de la distribution des scores du GENDER index selon le sexe, les deux concepts se sont avérés semblables, mais indépendants. La proportion de femmes était différente selon les différents sous-groupes formés grâce aux tertiles du GENDER index (*p* < 0,0001). La validité conceptuelle a aussi été examinée en mesurant les associations entre les scores du GENDER Index et différentes variables liées au genre et identifiées à priori telles que le fait de choisir certains aliments en raison d’une préoccupation pour son poids corporel (*p* < 0,0001), le fait que les soins à donner aux enfants soient identifiés comme principale source de stress (*p* = 0,0309), ou la capacité à faire face à des problèmes inattendus et difficiles (*p* = 0.0375).

**Conclusion:**

Le GENDER Index pourrait être utile pour renforcer la capacité des chercheurs à effectuer une analyse fondée sur le genre dans des populations de travailleurs grâce aux données de l’ESCC.

## Introduction

Despite growing attention given to the importance of considering sex and gender in health research (Johnson et al. 2009; Day et al. 2017; McGregor et al. 2016; Pilote and Humphries 2014), these terms are still used inconsistently and interchangeably in the literature (Vissandjee et al. 2016; Boerner et al. 2018). Whereas sex refers to a set of biological attributes and is associated with physical and physiological features (CIHR 2018), gender can be defined as socially constructed roles, behaviours, expressions, and identities of girls, women, boys, men, and gender diverse people (CIHR 2018). Gender is an important construct to examine as it influences how people perceive themselves and each other, how they act and interact, and the distribution of power and resources in society (CIHR 2018).

Measurement of biological sex is relatively straightforward (male, female, intersex) and is usually included as a variable in clinical and epidemiological studies (Vissandjee et al. 2016). As for gender, some validated self-report indexes are available for the measurement of selected gender constructs in prospective studies (e.g., gender roles, identity, relations) (Nanda 2011; McHugh and Hanson Frieze 1997; Shulman et al. 2017; Kachel et al. 2016; Bem 1974). However, many large administrative databases or surveys do not include gender measures, mostly because it has not been planned from the outset. The secondary analysis of such data sources is, nonetheless, indispensable to enriching our understanding of health trajectories, healthcare utilization, and real-world risks and benefits of drugs among large populations (Schneeweiss and Avorn 2005; Tamblyn et al. 1995; Bernatsky et al. 2013; Hashimoto et al. 2014).

Even if researchers have the opportunity to include various gender-related variables in multivariate modeling of various health outcomes (examples of gender-related variables include time spent on child care, occupation, number of working hours, types of leisure activities, stress (Bekker 2003)), the calculation of a single composite score is a statistically efficient option (Glynn et al. 2006). Various approaches have been proposed to derive composite gender indexes using existing data (Lippa and Connelly 1990; Pelletier et al. 2015; Smith and Koehoorn 2016; Canadian Institutes of Health Research 2017). For example, Smith and Koehoorn (2016) assigned a numerical value to each response category of four gender-related variables available in the Canadian Labour Force Survey (responsibility for caring for children, occupation, number of hours of work, and level of education). They then created a gender score by summing these variables (Smith and Koehoorn 2016). Although the proposed approach was simple and the resulting gender index showed face validity and sensitivity to change, the method was subjective since assumptions and categorizations were made about what answers were more feminine or more masculine. In contrast, other statistical approaches may be used to minimize researchers’ subjectivity surrounding the processing of variables for the computation of a composite index. Using gender-related variables available in the GENESIS-PRAXY cardiovascular study, Pelletier et al. (2015) derived a gender score using a principal component analysis and a logistic regression model where sex served as the dependent variable for the calculation of a propensity score.

The Canadian Community Health Survey (CCHS) is a rich source of detailed self-reported information about the health status, health risk factors, and use of healthcare services among Canadians (Statistics Canada 2012), and its secondary analysis is of great value for research purposes (Sanmartin et al. 2016; Raina et al. 1999; Yergens et al. 2014). However, the CCHS does not contain questions about gender, thus limiting the usefulness of the survey data for researchers interested in the topic and its relation to the health of Canadians. Moreover, to the best of our knowledge, a composite gender index has not been derived using the CCHS data. The aim of this study was to create and validate a composite gender index, namely the GENDER Index, using selected variables available from the CCHS.

## Methods

### Data source

The current study was conducted using the TORSADE Cohort (TrajectOiRes SAnté - Données Enrichies), an infrastructure of the Quebec SUPPORT Unit (Support for People and Patient-Oriented Research and Trials). This database was created with the aim of better understanding healthcare trajectories associated with ambulatory care sensitive conditions. This cohort of 60,791 individuals living in the province of Quebec results from the linkage between data from Statistics Canada’s CCHS (questionnaires 2007–2008, 2009–2010, and 2011–2012) and those of the administrative longitudinal databases (1996 to 2016) held by the Régie de l’assurance maladie du Québec (RAMQ). Authorization was granted by the Commission d’accès à l’information du Québec before data linkage and approval was obtained from concerned university Research Ethics Boards.

The CCHS collects data about the health of individuals of at least 12 years of age living in the ten Canadian provinces and the three territories (probability sampling) (Statistics Canada 2012). Not included are individuals living on Aboriginal reserves, full-time members of the Canadian Forces, institutionalized individuals, or persons living in the Quebec regions of Nunavik and Terres-Cries-de-la-Baie-James (altogether less than 3% of the Canadian population). CCHS response rates are high (69.8–78.9% depending on the cycle (Sanmartin et al. 2016)), response rates are similar in the province of Quebec vs the whole of Canada (Statistics Canada 2010a), and test-retest reliability of the answers to several questions has been well demonstrated (Raina et al. 1999). The TORSADE cohort contains data of all CCHS participants who accepted to share their data with Quebec’s Statistics Institute and agreed to data linkage (92.8% of CCHS participants) (Institut de la statistique du Québec 2018). In the 2007–2008, 2009–2010, and 2011–2012 CCHS questionnaires, biological sex was measured as a dichotomous variable (male vs female) without a “do not know” option.

For the following reasons, only the CCHS variables were considered for the creation of the GENDER Index: (1) the CCHS database is much richer than the Quebec administrative ones in terms of potentially gender-related socio-economic information, (2) the calendar date of the CCHS questionnaire is often defined as the index date in studies using the TORSADE Cohort, which makes it more logical to calculate gender scores at the date of completion of the questionnaire, and (3) Quebec administrative databases are not always available to researchers in other Canadian provinces who work with CCHS data.

### Identification of gender-related variables

A screening for potentially gender-related CCHS variables was achieved based on the following: (1) the Multi-Facet Gender and Health Model (Bekker 2003), (2) the different gender constructs proposed by Johnson et al. (2009) (gender roles, gender identity, gender relations, and institutionalized gender), (3) a review of variables considered in studies that derived composite gender indexes using other administrative/existing survey data (Lippa and Connelly 1990; Pelletier et al. 2015; Smith and Koehoorn 2016). Three members of the study team (one with expertise in the field of sex and gender, two in the field of epidemiology and biostatistics) discussed and reached a consensus about relevant CCHS variables. A very conservative approach was used at this point and all variables potentially relevant were considered (see Table 1). However, to be eligible, variables had to be measured in the three cycles of the CCHS (questionnaires 2007–2008, 2009–2010, and 2011–2012), be collected in the Canadian province of Quebec, and have ≤ 15% missing values (cut-off for which missing values can be considered problematic (Fox-Wasylyshyn and El-Masri 2005)). Although healthcare resources and medication use can be gender-related (Bekker 2003), they were not retained for the creation of the GENDER Index because such variables are expected to be important outcomes of future epidemiological and pharmacoepidemiological research projects conducted using the TORSADE Cohort or CCHS data.

**Table 1:**
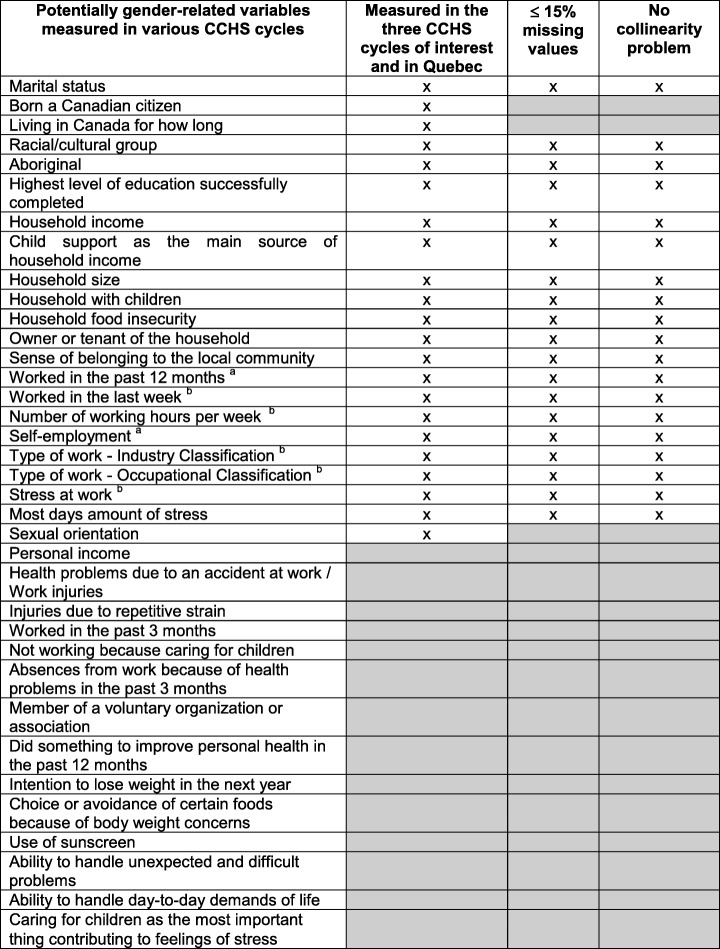

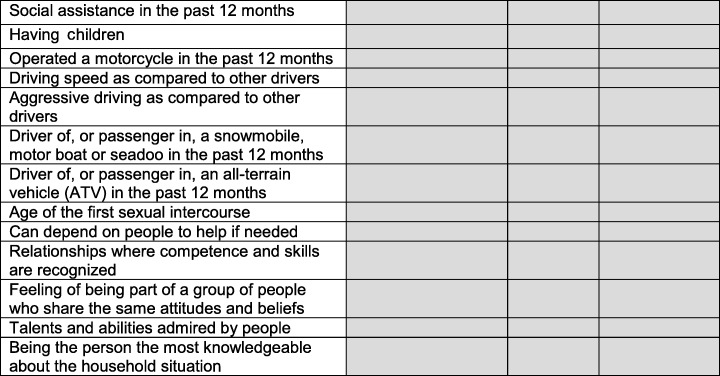
Candidate variables for deriving the GENDER Index

The selection process led to a total of 19 candidate variables (Table 1). According to the literature, occupational characteristics are important gender-related variables to be considered in the creation of a gender index (Bekker 2003) and CCHS work-related variables are measured among participants aged 18–50 years. A back-and-forth process between our modelization and our results also suggested that occupational characteristics were also among the most important variables for the creation of the GENDER Index. For these reasons, the current study was conducted in the sample of participants employed in the past 12 months and aged 18–50 years. Aboriginal status was not included in the GENDER Index because none of the participants reported being Aboriginal.

### Creation of the GENDER Index

The GENDER Index was derived using a propensity scoring approach. This approach was inspired by the work of Pelletier et al. (2015) that was endorsed by the Canadian Institutes of Health Research (CIHR) in their online training modules on integrating sex and gender in health research (Canadian Institutes of Health Research 2017).

The GENDER Index composite scores were derived following these steps: First, collinearity was explored among all the candidate variables using variance inflation factors (VIF) (O’Brien 2007) and parametric or non-parametric independent samples tests (according to the type and distribution of variables). All VIF values respected cut-offs suggested for detecting multicollinearity (VIF greater than 5 or 10 (Vatcheva et al. 2016)). Since none of the variables explained entirely or most entirely another variable, no exclusions were applied at this point (Table 1). All candidate variables were then included as independent variables (covariates) in a multiple logistic regression model for which biological sex served as the dependent variable (female = 1, male = 0). In such a multiple regression model, a propensity score can be derived for each participant, which can be defined as the conditional probability for a participant to have the outcome of interest given his observed covariates. Propensity score values can be added to the dataset as a new variable by adding a simple output command when running SAS® proc logistic. In our study, the probability of each respondent to be a female given the estimates from the logit model was calculated, which formed the propensity score and was included as a new variable in the dataset (i.e., the GENDER Index score). Higher scores on the 0–100 GENDER Index can be interpreted as a higher level of characteristics associated with being female/having more feminine characteristics.

It should be acknowledged from the outset that using biological sex as the dependent variable in our regression model can be criticized because it merges the related but different concepts of sex and gender (Johnson et al. 2009). However, previous authors showed that even if biological sex was used to create a gender score (Lippa and Connelly 1990; Pelletier et al. 2015), the two variables appeared as related but partly independent in the analysis (e.g., great variability of gender scores within each sex). Pelletier et al. (2015) also argued that defining gender-related variables as psychosocial variables that differ between males and females is concordant with the literature which often refers to gender as roles, attitudes, opportunities, and expectations held by males and females.

### Validity analysis

In addition to the calculation of descriptive statistics to summarize respondents’ characteristics, analyses were undertaken to explore the validity of the GENDER Index among the TORSADE Cohort. Face validity is the extent to which the items/components of an index look as though they are an adequate reflection of the construct to be measured (Mokkink et al. 2010). This property was examined by measuring the associations between each gender-related variable included in the GENDER Index and the gender score itself using univariate linear regression analyses. Construct validity can be defined as the extent to which the scores of an index are consistent with hypotheses (e.g., internal relationships, relationships with scores of other instruments, differences between relevant groups) based on the assumption that the index validly measures the construct under study (Mokkink et al. 2010). Construct validity was thus assessed by (1) comparing the distribution of GENDER Index scores between males and females using overlapping histograms, (2) comparing the proportion of females between groups categorized according to the GENDER Index scores tertiles (division of the ordered scores distribution into three parts, each containing a third of the population), and (3) examining the associations between presumed gender-related variables that were not included in the creation of the GENDER Index and GENDER Index scores using univariate linear regressions (i.e., choice or avoidance of certain foods because of body weight concerns, ability to handle unexpected and difficult problems, caring for children as the most important thing contributing to feelings of stress). These variables deemed to be gender-related were not included in the GENDER Index because they were not available for all CCHS cycles. Finally, in order to test the impact of various methodological approaches on the validity of the GENDER Index, sensitivity analyses were conducted by reducing the number of variables to be included in the multiple logistic regression model used to create the GENDER Index using a backward elimination technique until all remaining variables had *p* values < 0.05 (an approach used by Pelletier et al. 2015). Data analyses were performed using SAS® (version 9.4, Cary, NC, USA). Appropriate CCHS sampling weights and bootstrap variance estimation procedures were used (Statistics Canada 2012).

## Results

Among the 60,791 individuals of the TORSADE Cohort, a total of 29,470 (48.24%) participants employed in the past 12 months and aged 18–50 years had no missing data for any of the variables included in the GENDER Index. Characteristics of the study sample are presented in Table 2.

**Table 2:** Demographic and other characteristics of the sample

Characteristics, *n* = 29,470	Weighed frequency ^b^	Proportion ^b^ (%)
Sum of weights *n* = 3,689,207 ^a^		
Age – mean ± SD	40.45	± 0.08
Sex		
Females	1,739,602	47.15
Males	1,949,605	52.85
Household size (number of people)		
1	551,317	14.94
2	121,148	32.83
3–4	1,577,424	42.76
≥ 5	349,218	9.47
Marital status		
Married	1,278,654	34.66
Living common-law	1,036,956	28.11
Single	1,028,001	27.87
Divorced	203,665	5.52
Separated	103,547	2.81
Widowed	38,383	1.04
Racial/cultural group ^c^		
White	3,405,682	92.31
Arab	78,132	2.12
Latin American	61,445	1.67
Asian	53,674	1.45
Other	90,274	2.45
Household food insecurity (past 12 months)		
Always had enough of the kinds of foods they wanted to eat	3,425,530	92.85
Enough to eat, but not always the kinds of food they wanted	239,665	6.50
Sometimes did not have enough to eat	19,374	0.53
Often did not have enough to eat	4638	0.13
Highest level of education successfully completed		
Grade 8 or lower (Québec: secondary II or lower)	130,794	3.55
Grade 9–10 (Québec: secondary III or IV)	431,396	11.69
Grade 11–13 (Québec: secondary V)	1,857,692	50.35
College/CÉGEP	654,905	17.75
Bachelor’s degree	425,151	11.52
University degree or certificate above bachelor’s degree	189,270	5.13
Household income before taxes (Canadian dollars)		
$0–$19,999	145,341	3.94
$20,000–$39,999	530,237	14.37
$40,000–$59,999	735,831	19.95
$60,000–$79,999	711,800	19.29
$80,000–$99,999	510,048	13.83
≥ $100,000	1,055,950	28.62

*SD* standard deviation

^a^Appropriate survey sampling weights and bootstrap variance estimation procedures were used in all analyses (Statistics Canada 2012)

^b^Unless stated otherwise

^c^None of the participants reported being Aboriginal (North American Indian, Métis or Inuit)

The multiple logistic regression model used to create the propensity scores (GENDER Index scores) and all variables that were considered are presented in Table 3. The categorization of gender-related variables led to a total of 43 dummy variables included in the model (*c* = 0.796). In regard to our sample size, it respects the recommended events per independent variable ratio of 10:1 (Harrell et al. 1996). Sensitivity analyses revealed that the number of variables to be included in the multiple logistic regression model was not affected by the backward elimination technique.

**Table 3:** Results of the multivariate logistic regression analysis used to create the GENDER Index in which biological sex served as the dependent variable (female = 1, male = 0)

Variables included in the gender score	Multivariate logistic regression model		
	OR ^a^	95% confidence interval	
Marital status			
Single	Reference		
Married	1.166	*1.028*	*1.322*
Living common-law	1.322	*1.164*	*1.502*
Widowed	2.543	*1.247*	*5.186*
Separated	1.354	*1.045*	*1.755*
Divorced	2.316	*1.921*	*2.793*
Racial/cultural group			
Non-white (others)	Reference		
White	1.190	0.960	1.474
Highest level of education successfully completed			
Grade 8 or lower (Québec: secondary II or lower)	Reference		
Grade 9–10 (Québec: secondary III or IV)	1.092	0.856	1.393
Grade 11–13 (Québec: secondary V)	1.279	*1.017*	*1.609*
College/CÉGEP	1.457	*1.148*	*1.849*
Bachelor’s degree	1.196	0.932	1.534
University degree or certificate above bachelor’s degree	0.931	0.692	1.253
Household income before taxes (Canadian dollars)			
$0–$19,999	Reference		
$20,000–$39,999	0.997	0.763	1.301
$40,000–$59,999	0.715	*0.547*	*0.934*
$60,000–$79,999	0.595	*0.454*	*0.781*
$80,000–$99,999	0.547	*0.408*	*0.733*
≥ $100,000	0.457	*0.345*	*0.605*
Child support as the main source of household income			
No	Reference		
Yes	3.304	0.023	465.152
Household size			
1	Reference		
2	1.250	*1.086*	*1.438*
3–4	1.306	*1.106*	*1.544*
≥ 5	0.969	0.754	1.245
Household with children (≤ 15 years old)			
No	Reference		
Yes	1.092	0.958	1.245
Household food insecurity (past 12 months)			
Always had enough of the kinds of foods they wanted to eat	Reference		
Enough to eat, but not always the kinds of food they wanted	0.982	0.809	1.194
Sometimes did not have enough to eat	1.157	0.591	2.265
Often did not have enough to eat	1.156	0.440	3.035
Ownership of the household			
Tenant	Reference		
Owner	1.298	*1.165*	*1.447*
Sense of belonging to the local community			
Very weak	Reference		
Somewhat weak	0.960	0.814	1.133
Somewhat strong	1.026	0.877	1.200
Very strong	0.898	0.737	1.095
Worked in the last week			
No	Reference		
Yes	0.654	*0.558*	*0.765*
Number of working hours per week			
< 35	Reference		
≥ 35	0.417	*0.376*	*0.463*
Self-employment			
No	Reference		
Yes	0.556	*0.490*	*0.630*
Industry classification—health care and social assistance sector ^b^			
No	Reference		
Yes	2.481	*2.100*	*2.931*
Industry classification—construction/manufacturing sectors ^b^			
No	Reference		
Yes	0.509	*0.447*	*0.579*
Occupational classification—health occupations/occupations in social science, education, government service, and religion ^b^			
No	Reference		
Yes	1.823	*1.557*	*2.134*
Occupational classification—trades, transport and equipment operators, and related occupations/occupations unique to primary ^b^ industry			
No	Reference		
Yes	0.107	*0.090*	*0.127*
Stress at work			
Most days at work are not at all stressful	Reference		
Not very stressful	1.314	*1.087*	*1.587*
A bit stressful	1.184	0.994	1.411
Quite a bit stressful	1.220	*1.003*	*1.483*
Extremely stressful	1.529	*1.166*	*2.003*
Most days amount of stress			
Most days are not at all stressful	Reference		
Not very stressful	1.615	*1.341*	*1.944*
A bit stressful	1.868	*1.571*	*2.220*
Quite a bit stressful	2.114	*1.752*	*2.551*
Extremely stressful	2.181	*1.641*	*2.898*

*OR* odds ratio

Italicized confidence intervals indicate statistically significant associations (the confidence interval does not include 1)

^a^OR > 1 indicates a higher level of characteristics associated with women/more feminine characteristics

^b^For the purpose of this study, industry and occupational classifications were recategorized according to occupations that most differ between sexes (Statistics Canada 2010b)

### Face validity of the GENDER Index

Results of univariate linear regression analyses measuring the associations between each variable included in the GENDER Index and the gender score itself are presented in Table 4. Associations (*p* < 0.05) were found for all variables except for ownership of the household (owner vs tenant), supporting the extent to which variables used to create the GENDER Index were relevant to the gender score. The six variables with the highest regression coefficients (*β*) were as follows: (1) having an occupation in the field of trades, transport, and equipment operators, related occupations, or occupations unique to primary industry, (2) receiving child support as the main source of household income, (3) working in an organization of the healthcare or social assistance sector, (4) having an occupation in the field of health, social science, education, government service, or religion, (5) working in an organization of the construction or manufacturing sector, (6) number of working hours per week.

**Table 4:** Associations between each variable included in the GENDER Index and the gender score itself

Variables included in the gender score ^a^	Univariate linear regression models		
	*β*	SE	*p* value
Marital status			
Single	Reference		
Married	− 0.002	0.006	0.7079
Living common-law	0.020	0.006	*0.0004*
Widowed	0.212	0.022	*< 0.0001*
Separated	0.047	0.012	*< 0.0001*
Divorced	0.171	0.010	*< 0.0001*
Racial/cultural group			
Non-white (others)	Reference		
White	0.043	0.012	*0.0006*
Highest level of education successfully completed			
Grade 8 or lower (Québec: secondary II or lower)	Reference		
Grade 9–10 (Québec: secondary III or IV)	0.037	0.013	*0.0054*
Grade 11–13 (Québec: secondary V)	0.139	0.012	*< 0.0001*
College/CÉGEP	0.222	0.014	*< 0.0001*
Bachelor’s degree	0.199	0.014	*< 0.0001*
University degree or certificate above bachelor’s degree	0.167	0.015	*< 0.0001*
Household income before taxes (Canadian dollars)			
$0–$19,999	Reference		
$20,000–$39,999	− 0.027	0.014	*0.0487*
$40,000–$59,999	− 0.089	0.014	*< 0.0001*
$60,000–$79,999	− 0.090	0.014	*< 0.0001*
$80,000–$99,999	− 0.100	0.014	*< 0.0001*
≥ $100,000	− 0.111	0.013	*< 0.0001*
Child support as the main source of household income			
No	Reference		
Yes	0.427	0.014	*< 0.0001*
Household size			
1	Reference		
2	0.011	0.006	*0.0442*
3–4	0.028	0.006	*< 0.0001*
≥ 5	− 0.034	0.010	*0.0006*
Household with children (≤ 15 years old)			
No	Reference		
Yes	0.029	0.005	*< 0.0001*
Household food insecurity (past 12 months)			
Always had enough of the kinds of foods they wanted to eat	Reference		
Enough to eat, but not always the kinds of food they wanted	0.025	0.012	*0.0307*
Sometimes did not have enough to eat	0.082	0.029	*0.0052*
Often did not have enough to eat	0.031	0.047	0.5110
Ownership of the household			
Tenant	Reference		
Owner	− 0.000	0.005	0.9887
Sense of belonging to the local community			
Very weak	Reference		
Somewhat weak	0.020	0.009	*0.0226*
Somewhat strong	0.029	0.008	*0.0003*
Very strong	− 0.022	0.010	*0.0360*
Worked in the last week			
No	Reference		
Yes	− 0.106	0.008	*< 0.0001*
Number of working hours per week			
< 35	Reference		
≥ 35	− 0.241	0.005	*< 0.0001*
Self-employment			
No	Reference		
Yes	− 0.119	0.006	*< 0.0001*
Industry classification—health care and social assistance sector			
No	Reference		
Yes	0.380	0.004	*< 0.0001*
Industry classification—construction/manufacturing sectors			
No	Reference		
Yes	− 0.300	0.004	*< 0.0001*
Occupational classification—health occupations/occupations in social science, education, government service, and religion			
No	Reference		
Yes	0.338	0.004	*< 0.0001*
Occupational classification—trades, transport and equipment operators, and related occupations/occupations unique to primary industry			
No	Reference		
Yes	− 0.468	0.002	*< 0.0001*
Stress at work			
Most days at work are not at all stressful	Reference		
Not very stressful	0.095	0.010	*< 0.0001*
A bit stressful	0.080	0.008	*< 0.0001*
Quite a bit stressful	0.118	0.008	*< 0.0001*
Extremely stressful	0.156	0.012	*< 0.0001*
Most days amount of stress			
Most days are not at all stressful	Reference		
Not very stressful	0.142	0.009	*< 0.0001*
A bit stressful	0.149	0.008	*< 0.0001*
Quite a bit stressful	0.193	0.008	*< 0.0001*
Extremely stressful	0.196	0.015	*< 0.0001*

Italicized *p* values indicate statistically significant associations (*p* < 0.05)

^a^Higher scores on the 0–100 GENDER Index can be interpreted as a higher level of characteristics associated with being female/having more feminine characteristics

### Construct validity of the gender index

**Fig. 1:**
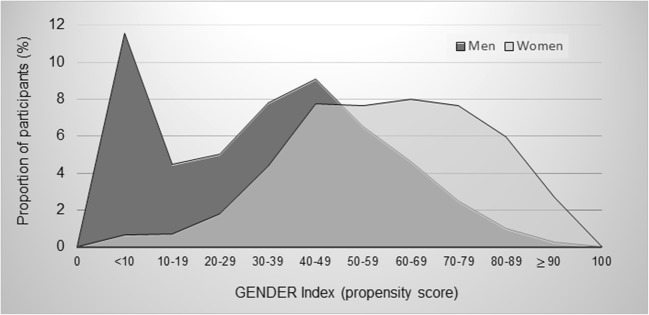
Distribution of GENDER Index scores in men and women. Higher scores on the 0–100 GENDER Index can be interpreted as a higher level of characteristics associated with being female/having more feminine characteristics (Created with Excel software)

The distribution of GENDER Index scores in males and females is represented in Fig. 1. According to this visual representation, sex and GENDER Index scores appeared related but partly independent (e.g., incomplete histogram overlap, variability of gender scores within each sex group). Differences were also found in the proportion of females between groups categorized according to the GENDER Index scores tertiles (tertile 1: 14.90% vs tertile 2: 36.84% vs tertile 3: 48.26%, *p* value < 0.0001).

Regarding associations between GENDER Index scores and presumed gender-related variables identified a priori and not included in the index GENDER Index, univariate linear regression models revealed that choosing or avoiding certain foods because of body weight concerns (*β* 0.046, *p* < 0.0001) and caring for children as the most important activity contributing to feelings of stress (*β* 0.048, *p* = 0.0309) were associated with higher GENDER Index scores (presumed to represent more feminine characteristics). A greater ability to handle unexpected and difficult problems (*β* excellent vs poor − 0.093, *p* = 0.0375) was associated with lower GENDER Index scores (more masculine characteristics).

## Discussion

To our knowledge, this is the first study to derive a composite gender index using CCHS data. Validity of an index can be defined as the extent to which all of the accumulated evidence supports the intended interpretation of the scores for the intended purpose (Streiner and Kottner 2014; AERA/APA/NCME 2014). Our results thus suggest that the GENDER Index could be useful to enhance the capacity of researchers using workers CCHS data to conduct gender-based analysis in the absence of self-reported gender measures.

The GENDER Index development was intended to maximize its face validity. Almost all variables included in the GENDER Index also appeared to be important when they were examined in relation to the total score. Variables most related to the total score (occupation, receiving child support as the main source of household income, and number of working hours per week) were consistent with variables retained by other authors when creating composite gender indexes (responsibility for caring for children, occupation, number of hours of work (Smith and Koehoorn 2016), and hours per week doing housework (Pelletier et al. 2015)). Using known-groups and convergent validity analytical approaches, various arguments towards the construct validity of the use of the GENDER Index are also provided.

The GENDER Index is a multidimensional composite score and was not intended to represent only one gender construct. When looking at the variables available in the CCHS and included in the index, some characteristics such as childcare responsibilities and type of work can relate to gender roles (behavioural norms applied to men and women) (Johnson et al. 2009). Race and interactions within social units can interact with gender relationships (how individuals interact with and are treated by others based on their ascribed gender) (Johnson et al. 2009). We can therefore argue that considering variables such as race and sense of belonging to the local community in the creation of the GENDER Index expands its multidimensional nature. Aspects related to institutionalized gender (how power and influence are distributed differently among men and women) (Johnson et al. 2009) were also represented through the inclusion of variables such as race, education, job limitations (e.g., stress at work), and access to resources such as money or food. Since marital status can be related to opportunities afforded to the genders (e.g., job opportunities) (Nadler and Kufahl 2014) and stress can be related to gender roles or gender identities (Jones et al. 2016; Eisler et al. 1988), such variables were also relevant to our work.

Gender is an important construct to enhance our understanding of health determinants, disease courses, and treatment outcomes. In fact, it can be associated with important aspects surrounding both communicable and chronic diseases, such as experience and expression of physical symptoms (e.g., pain (Boerner et al. 2018)), health behaviours (e.g., vaccination (Vamos et al. 2018), treatment adherence (Sajatovic et al. 2011), alcohol or drug use (Lye and Waldron 1998)), coping strategies (Spendelow et al. 2018), and expectations (Bekker 2003). Using their composite gender score, Pelletier et al. (2015) found that, independently from biological sex, gender was associated with cardiovascular risk factors such as hypertension, diabetes, family history depressive symptoms, and anxious symptoms. The same team also found an association between gender scores and serious health outcomes such as recurrence of acute coronary syndrome (Pelletier et al. 2016).

When analyzing administrative databases or existing survey data, researchers have the possibility to identify various gender-related variables and include them in multiple regression modeling of various health outcomes. However, the use of a composite gender score offers advantages. Such scores can be used for adjustment in multiple regression models, matching, and subgroup stratification (using measures of position such as tertiles) in order to better control confounding variables in observational studies (Glynn et al. 2006). As compared with the use of a set of gender-related variables, they provide greater statistical power by reducing the number of covariates included in multiple regression models, offer the possibility to test interaction terms, and reduce multiple comparisons (Glynn et al. 2006; Song et al. 2013).

### Limitations

First, it was not possible to examine the validity of the GENDER Index by comparing it with an existing validated gender assessment instrument since the CCHS does not include such a tool. It is also important to underline that the validity of the index should be further investigated in different populations (e.g., validation subsample or more recent CCHS cycles). Another limitation of our study has to do with the generalizability of the GENDER Index to age groups not included in the current study. Because occupational characteristics were important gender-related variables to be considered in the creation of a gender index, the GENDER Index could only be calculated in workers. Although this aspect is a major threat to our study’s external validity, the GENDER Index could be useful for many researchers (e.g., in the field of occupational health). Further studies should explore the validity of indexes that can be calculated without considering occupational characteristics.

## Conclusions

This investigation provides a methodological example for researchers who wish to conduct gender-based analysis of existing databases when self-reported gender data are unavailable. Despite the limitations of our study, the results support the value of the GENDER Index as a new tool to enhance the capacity of researchers using CCHS data to conduct gender-based analysis among populations of workers.
